# Components of the transitional care model (TCM) to reduce readmission in geriatric patients: a systematic review

**DOI:** 10.1186/s12877-020-01747-w

**Published:** 2020-09-11

**Authors:** Nadine Morkisch, Luz D. Upegui-Arango, Maria I. Cardona, Dirk van den Heuvel, Martina Rimmele, Cornel Christian Sieber, Ellen Freiberger

**Affiliations:** 1 Bundesverband Geriatrie e.V, Berlin, Germany; 2 Institute of Medical Psychology and Medical Sociology, University Hospital of RWTH Aachen, Aachen, Germany; 3 Institute of Biomedicine of Aging, Nuremberg, Friedrich-Alexander-University Erlangen-Nuremberg, Kobergerstr. 60, 90408 Nuremberg, Germany; 4 Kantonspital Winterthur/Swiss, Winterthur, Switzerland

**Keywords:** Systematic review, Transitional care, Transitional care model, Geriatric patients, Readmission

## Abstract

**Background:**

Demographic changes are taking place in most industrialized countries. Geriatric patients are defined by the European Union of Medical Specialists as aged over 65 years and suffering from frailty and multi-morbidity, whose complexity puts a major burden on these patients, their family caregivers and the public health care system. To counteract negative outcomes and to maintain consistency in care between hospital and community dwelling, the transitional of care has emerged over the last several decades. Our objectives were to identify and summarize the components of the Transitional Care Model implemented with geriatric patients (aged over 65 years, with multi-morbidity) for the reduction of all-cause readmission. Another objective was to recognize the Transitional Care Model components’ role and impact on readmission rate reduction on the transition of care from hospital to community dwelling (not nursing homes).

**Methods:**

Randomized controlled trials (sample size ≥50 participants per group; intervention period ≥30 days), with geriatric patients were included. Electronic databases (MEDLINE, CINAHL, PsycINFO and The Cochrane Central Register of Controlled Trials) were searched from January 1994 to December 2019 published in English or German. A qualitative synthesis of the findings as well as a systematic assessment of the interventions intensities was performed.

**Results:**

Three articles met the inclusion criteria. One of the included trials applied all of the nine Transitional Care Model components described by Hirschman and colleagues and obtained a high-intensity level of intervention in the intensities assessment. This and another trial reported reductions in the readmission rate (*p* < 0.05), but the third trial did not report significant differences between the groups in the longer follow-up period (up to 12 months).

**Conclusions:**

Our findings suggest that high intensity multicomponent and multidisciplinary interventions are likely to be effective reducing readmission rates in geriatric patients, without increasing cost. Components such as type of staffing, assessing and managing symptoms, educating and promoting self-management, maintaining relationships and fostering coordination seem to have an important role in reducing the readmission rate. Research is needed to perform further investigations addressing geriatric patients well above 65 years old, to further understand the importance of individual components of the TCM in this population.

## Background

Demographic changes are taking place in most industrialized countries. In Germany in 2014, the population 66–99 years of age accounted for 20% (16.1 million) of the total population [1, 2]. It is estimated that by 2060, this group of older people will account for 33% (22.7 million) of the total population in Germany [1]. These demographic changes are predicted to produce a massive burden on Macro and Micro level. On the population level (Macro level) politics have to decide about financial aspects of the public health care system as well as ethical considerations, and on individual level (Micro level) intrinsic capacity as formulated by the WHO (2015) will play a major role in healthy aging [3].

Geriatric patients are defined by the European Union of Medical Specialists as aged over 65 years, having “a high degree of frailty and active multiple pathology” [4] and often multi-morbidity [5]. “Multi-morbidity” as defined by the WHO and the UK National Institute for Health Care Excellence (NICE) is multiple long-term health conditions (2 or more), which require complex and ongoing care [6, 7]. A prevalence of multi-morbidity in the older population has been reported as ranging from 55 to 98% [8, 9]. Multi-morbidity is associated with poorer quality of life, loss of function, polypharmacy, and care duplication as well as inconsistencies [10, 11]. A recent meta-analysis demonstrated that multi-morbidity increased the risk of death in geriatric patients [12]. The management of care for geriatric patients with multiple chronic conditions is often fragmented among health care practitioners (general vs. specialists) with poor handoffs after exacerbation of their conditions (emergency department to community dwelling, hospital to community dwelling, hospital to skilled care facility) [7, 13]. The complexity of multi-morbidity in the context of frailty or dementia in connection with polypharmacy puts a major burden on the geriatric patients, their family care givers and the public health care system [7, 10].

To counteract negative outcomes (e.g. hospitalization or re-admission to hospital), for this population of geriatric patients with multi-morbidity, and to maintain consistency in care between the hospital and community dwelling, the topic of transitional of care as both an area of research and practice has emerged over the last several decades [14–16]. The WHO defines transitions of care as “when a patient moves to, or returns from, a particular physical location or makes contact with a health care professional for the purposes of receiving health care. This includes transitions between community dwelling, hospital, residential care settings and consultations with different health care providers in out-patient facilities” [6]. Transitional care is a set of strategies and services offered to improve care transitions, and aspects of safe and timely passage of patients between levels of health care and across care settings and are time limited to these situations [17–19].

Allen and colleagues [20], suggest that for a successful transition, the essential interventions might be: discharge assessment and care planning, provider communication, preparation of the person and caregiver for transition of care, medication reconciliation in transition, community-based follow-up, and patient education in self-management [21–23]. Where, for example, interventions such as medication reconciliation have been linked to reducing adverse events associated with non-adherence to medication after hospital discharge [21]. The reduction of adverse events has also been related to interventions with a multidisciplinary approach as well as communication between health professionals during the transition from patient to home [24]. The Transitional Care Model (TCM), a multicomponent, nurse-led intervention has been tested in the U.S. and has consistently shown that the intervention which is provided on average for 60 days (range 1–3 months) can increase time to first re-hospitalization or death, decrease the number of hospitalization readmissions and number of days hospitalized, decrease costs and improve patient reported outcomes [17, 25–27]. The TCM features a hospital to community dwelling intervention with nine core components. The nine components are not necessarily performed one after the other but in combination. Therefore, tailoring the intervention components (e.g. varying intensity, different combinations) to meet the needs of the patient and their family caregiver are essential to achieve best results for the patients (Table 4) [31].

A reduction of hospital readmission rates (from 12 to 75% reduction) has been reported in some randomized studies with the use of interventions approaching patient education, pre-discharge evaluation and domiciliary patient-centered care [24, 32]. In general, these interventions have been described as part of transitional care, a set of activities aimed at patients with heightened risk of readmission. These groups of people comprise particularly people in vulnerable conditions such as older people, children and those suffering from chronic affections that require complex healthcare [20, 33]. These interventions promote the safe and appropriate transfer of patients from one setting to another, mostly from hospital to community dwelling, without an interruption of care. Transitional care interventions are mainly nurse-led interventions, but these can be also adapted to be carried out by other trained health professionals, which might contribute to improve the outcomes after hospital discharge [34–36].

### Objectives

Previously, some systematic reviews had evaluated randomized controlled trials (RCTs) in order to assess the effectiveness of transitional care interventions with older people. These studies were done with different focuses, e.g. special diseases such as stroke, heart failure, chronic obstructive pulmonary disease (COPD) and asthma, among other chronic illnesses [37, 38], outcome parameters other than readmission rate as mortality, activities of daily life (ADLs), functional status, mental status, patient satisfaction and caregiver burden, among others [20, 37], or special range of age for example eligibility criteria ≥60 or ≥ 65 years of age [20, 39]. However, to date, there is no published systematic review focusing on which components of the TCM are used in RCTs compared to any type of usual care for an all-cause readmission rate reduction in the geriatric patient population (age over 65 years). Since geriatric patients usually suffer from a combination of multiple health conditions, we believe that a broader concept of reviewing is needed [4, 38, 40]. In addition, as this very specific population of geriatric patients is predicted to grow rapidly over the next decades, effective strategies to reduce hospital readmission rates in this group of people will become critical to meeting their needs, therefore a systematic review is needed on the effectiveness of the TCM addressing:

- hospital setting (and not Emergency Department (ED)) and community dwelling (and not nursing homes),
- targeting multi-morbidity (and not disease specific).

There will be openness about the diseases to be studied, but meeting the definition of the geriatric patient by the EUMS [4] is mandatory. Demographic changes with higher percentage of a geriatric population represent a challenge for public health systems, which can be overcome with the support of comprehensive approaches and strategies that contribute to a successful transition from hospital to community dwelling.

For a better understanding of the transitional care literature and to address this gap, the present systematic review was conducted. Therefore, the aim was to identify and summarize the components of the Transitional Care Model implemented with geriatric patients to reduce all-cause readmissions as well as to recognize the TCM-components’ role and impact on readmission rate reduction on the transition of care from hospital to community dwelling [31].

## Methods

### Protocol and registration

The systematic review was carried out according to the Preferred Reporting Items for Systematic Reviews and Meta-Analyses (PRISMA) guidelines [41]. In order to have a guide while conducting the systematic review, an a priori detailed protocol was developed, which described the review’s rationale, objectives, and planned methods. This protocol was registered in PROSPERO (International Prospective Register of Systematic Reviews) and is available at:

http://www.crd.york.ac.uk/PROSPERO/display_record.php?ID=CRD42018084604

### Eligibility criteria

A standardized form was developed and used for eligibility screening as well as for data extraction of the identified included studies. The contents of the standardization were as follows:

#### Inclusion criteria

Only randomized controlled trials (RCT) published in either English or German language were included. The review included geriatric patients explicitly older than 65 years with comorbidities in order to comply with the European Union of Medical Specialists definition of a geriatric patient [4] and who were hospitalized due to an acute or chronic health condition. The initial sample size was set to ≥50 participants per group. Studies were only included if all study participants also had been discharged from hospital to community dwelling.

The intervention tested in the trials required the inclusion of the transition process from hospital to community dwelling, and at least one component of the TCM components at pre-discharge and one at post-discharge has to be described in detail (according to Hirschman and colleagues; see Table 4) [31]. Moreover, the duration of the recommended actions and interventions had to last for at least 30 days but no more than one year. Furthermore, only those studies that described their intervention protocol in detail were considered for inclusion.

#### Exclusion criteria

Trials with participants transferring from hospital to nursing homes or to any type of care other than the participant’s home were excluded. Transitions from ED stays to community dwelling were excluded. In addition, trials with interventions shorter than 30 days and/or with patients’ ≤ 65 years old were excluded.

### Outcome measures

Studies with the primary or secondary outcome all-cause readmission rate, defined as the number of study participants in each group hospitalized for any reason, were included in the review.

Other outcomes reviewed in this analysis, but not required, included activities of daily living, quality of life, changes in functional status, participation in ADLs and life roles, level of care, nutritional status, wound healing, death during the follow up and cost of care.

### Search strategy and study selection

A search strategy was developed in consultation with a librarian of the scientific medicine library of the Friedrich-Alexander University, Nuremberg - Germany. The search was carried out using the MEDLINE, CINAHL and the PsycINFO databases from January the 1st 1994 through November the 27th 2017, as well as The Cochrane Central Register of Controlled Trials (CENTRAL) (The Cochrane Library) from January the 1st 1994 through December 2019. English keywords were used for the electronic search (for the specific terms used in the search strategy, [see Additional file 1] or can be obtained from the corresponding author).

Furthermore, in order to identify further published, unpublished and ongoing trials, which were not available in the named electronic databases, a manual identification of articles in English or German in other sources until November the 28th 2017 was performed [see Additional file 2]. Additionally, a screening of relevant bibliographies of articles and books until November the 28th 2017 was carried out.

An interactive team of two reviewers (NM, and MIC/EF), at the two different institutions (an author from one center and two authors from the other center) performed the initial screening of titles and abstracts and the subsequent assessment for eligibility of retrieved full texts independently. Any disagreement or potential discrepancies in double coding were resolved through discussion with a third review author (MR).

### Data collection process

The data extraction from the articles included: study design, sample size, sample characteristics (i.e. age, gender and diagnosis, among others), study setting, TCM components used, hospital readmission rate with follow-up period, secondary outcomes, type of healthcare professionals involved in the intervention, and adverse events. Each component (Cp) of the TCM was classified in the pre-discharge and post-discharge phases. The TCM components were as follows: Cp1: screening, Cp2: staffing, Cp3: maintaining relationships, Cp4: engaging patients and caregivers, Cp5: assessing/managing risks and symptoms, Cp6: education/ promoting self-management, Cp7: collaborating, Cp8: promoting continuity and Cp9: fostering coordination [31]. In the post-discharge phase two TCM components (Cp 1: screening and Cp 2: staffing) were not included, since these are only used in the pre-discharge phase. Additional information regarding the statistical power of the trials was collected, such as sample size of each group and the *p* value used to evaluate statistical significance. In addition, to estimate the magnitude of the difference between the groups, percentage differences were calculated by dividing the absolute percentage difference between the groups by the percentage of the control group.

Missing information was requested from study authors. The authors were contacted three times. If there was no reply, the study was excluded. In the case of studies with analyses based on the same sample and intervention study, the most complete and/or most recent article was selected for the review while the other studies were excluded.

### Assessment of internal and external validity

Once the final studies were chosen, the two reviewers independently used the Cochrane Risk of Bias Tool to assess the internal validity of eligible studies [42, 43]. This assessment tool was used to evaluate methods of randomization, treatment allocation concealment, blinding of assessors, completeness of outcome data. A review team of five persons (LDU/NM, and MIC/MR/EF) forming an interactive team of two reviewers (one of each center) determined if the information presented in the articles about the trials had a low, high, or unclear risk of bias.

Additionally, external validity was assessed in order to identify accordance with everyday practice and clinical relevance of the included studies. The checklist proposed by Bornhoft and collaborators was used to perform this qualitative evaluation [44]. This checklist evaluates aspects such as study population assessment, intervention and control actions assessment, outcome measurements, results and evaluation assessments, and study design and setting. Moreover, precision of effect estimates and directness of the body of evidence were additionally included.

Disagreements between the two interactive review authors (e.g. NM and MIC) over the risk of bias in internal and external validity of particular trials were resolved by discussion, with involvement of a third review author (LDU or MR).

### Synthesis of results

A qualitative synthesis of the findings from the included studies is provide in this review, structured around the primary outcome reduction of readmission rate, type of TCM components applied and relevant modifications, the target population characteristics, the healthcare profession or the multi-professional team involved on the intervention.

Furthermore, in order to describe in more detail, the implementation of the TCM components considered in the selected studies, a systematic assessment of the interventions intensities was performed. Thus, seven parameters guided this assessment according to Verhaegh and collaborators [38] and Vedel and colleagues [45]. The obtained points in each of the seven parameters were added up to find the total score of intervention intensity. Thus, a trial was considered High-intensity when it scored 22–31 points; Moderate-intensity = 11–21 points; and Low-intensity = 1–10 points [see Additional file 3].

## Results

### Study selection

**Fig. 1:**
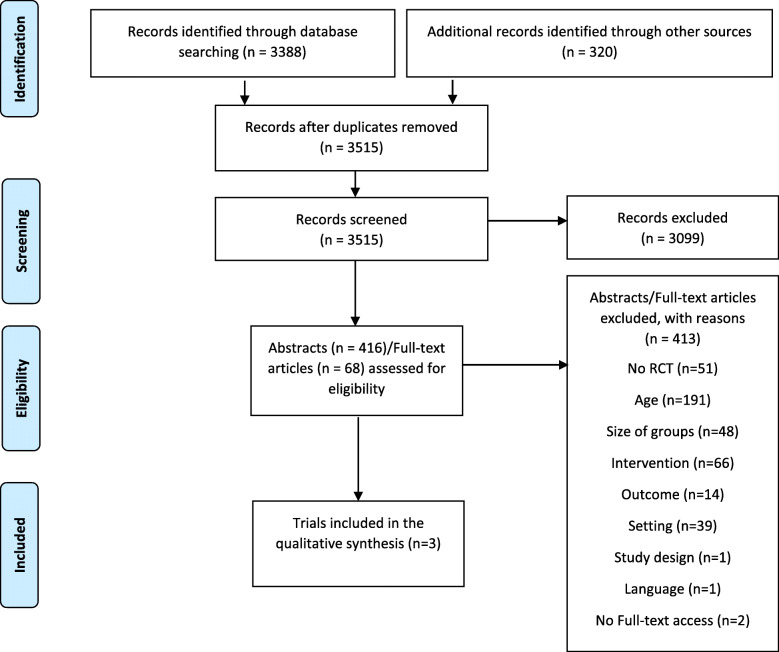
PRISMA flow diagram for trials included and excluded from the systematic review [41]

In total, 3388 articles from electronic search and 320 articles from hand search were found. After removing duplicates, 3515 articles were yielded. A total of 3099 titles were excluded due to not meeting eligibility criteria. Of the remaining 416 articles 348 had to be excluded upon abstract revision. Reasons for exclusion were e.g. not an RCT, participant age (≤65 years old), or sample size (< 50). Therefore, 68 full-text reviews were carried out, with a total of three trials adhering to all the established inclusion and exclusion criteria (Fig. 1). These three were analyzed in detail and were included in the present review. The studies were carried out in Australia [28], in Spain [29] and in the USA [30]. In the case of Rich et al. [30] and López Cabezas et al. [29], additional information related to the characteristics of the studies was also obtained through direct contact via email to the authors. Rich recommended the revision of an article of the same trial, previously published by the same authors [46]. López Cabezas confirmed by email that although age over 65 was not a criterion for inclusion in the study, all included participants were > 66 years old.

### Characteristics of participants and intervention

The participants of the included studies fulfilled the criteria of the European Union of Medical Specialists definition of geriatric patients, which corresponds to patients aged over 65 years and with comorbidities [4]. The mean age across intervention groups was 78.5 (±2.8) and across control groups 78.4 (±2.3). 62.2% were female in the intervention groups, and 60.6% in the control groups (Table 1). The study population of López Cabezas and colleagues [29] and Rich and colleagues [30] consisted of participants with heart failure and comorbidities e.g. hypertension or diabetes. Therefore, the participants were multi-morbid and can be described geriatric. The inclusion criteria of the sample in the study of Clemson and colleagues [28] were not defined on the basis of a specific diagnosis.

**Table 1:** Study characteristics

Study ID	Size of groups, n		Drop outs (%)		Age, Mean (SD)		Sex, N female (%)			
	Intervention group	Control group	Intervention group	Control group	Intervention group	Control group	Intervention group	Control group	Diagnosis	Profession
Clemson 2016 [28]	198	202	15%	16%	80.2 (6.4)	80.7 (5.7)	118 (59.6)	129 (63.9)	Not specified	Occupational therapists
López Cabezas 2006 [29]	70	64	nr^a^	nr^a^	75.3 (8.4)	76.1 (9.4)	41 (58.6)	34 (53.1)	Heart failure	Pharmacists
Rich 1995 [30]	142	140	nr	nr	80.1 (5.9)	78.4 (6.1)	96 (68.0)	83 (59.0)	Heart failure	Multidisciplinary team
N	410	406			78.5 (2.8)	78.4 (2.3)	255 (62.2)	246 (60.6)		

*SD* standard deviation, *nr* not reported

^a^ A general value of loss to follow-up of 9.6% for both groups was reported

The length of the interventions ranged from three months [30], to 12 months [29] post-discharge. Trained therapists providing the intervention included different health care professionals, particularly occupational therapists (OTs) in Clemson and colleagues’ study, pharmacists in López Cabezas and colleagues’ study, and a multi-professional team in Rich and colleagues’ study [28–30] (Tables 1 and 2).

**Table 2:** Study results on hospital readmission rate

Study ID	Intervention period	Hospital Readmission rate				
		Length of follow-up	Intervention group (%)	Control group (%)	Difference^**a**^ (%)	p value*
Clemson 2016 [28]	1 month	3 months	23.5%	21.9%	7.3	0.46
López Cabezas 2006 [29]	2 months	2 months	11.4%	25.0%	−54.4	< 0.05
	6 months	6 months	24.3%	42.2%	−42.4	< 0.05
	12 months	12 months	32.9%	48.4%	−32.0	nr
Rich 1995 [30]	3 months	3 months	28.9%	42.1%	−31.3	< 0.05

* Significant difference was defined as *p* < 0.05; nr: not reported; ^a^Percent differences of the readmission rates between the control group and the intervention group

In relation to the loss to follow-up, Clemson and colleagues [28] reported a loss to follow up of 15% in the control group receiving usual care and 16% in the intervention group receiving transitional care (Table 1). For their part, López Cabezas and colleagues [29] have only reported a general value of loss to follow-up of 9.6% for both groups.

### Primary outcome: reduction of readmission rate

#### Length and intensity of intervention

Rich and colleagues [30] observed a significant difference in the readmission rate of the participants from the control (42.1%) compared with the participants from the intervention group (28.9%) (CI 95%: 2.1 to 24.3, size of the percentage difference: − 31.3%, *p* = 0.03) (Table 2). The authors also described a greater occurrence of multiple hospital readmissions in the control (16.4%) versus the intervention group (6.3%) (*p* = 0.001). In addition, they carried out a long-term evaluation of the readmission rate reduction during the 9-month follow-up after the intervention was withdrawn, where a persistent readmission reduction in the heart failure group was observed (80 vs. 57, *p* = 0.08).

In the case of Clemson and colleagues [28], overall, no statistical significance differences between the percent of readmission of both groups at three months of follow-up were reported (control group: 21.9 vs intervention group: 23.5, size of the percentage difference: 7.3%, *p* = 0.46). The authors reported for the control group a percentage of 21.9 unplanned readmissions. This was corrected by the authors of the present systematic review using the data published by Clemson that corresponded to 20.9% (37 unplanned readmissions/ 177 N of the control group). Therefore, the percentage difference was estimated using the value 20.9% (control group: 20.9 vs intervention group: 23.5, size of the percentage difference: 12.4%).

Regarding the López Cabezas and colleagues’ trial [29], it was reported that no significant differences between the groups at 12 months of follow-up (control group: 48.4% vs intervention group: 32.9%, size of the percentage difference: − 32%, value p not reported by the authors) existed. On the other hand, the authors found significant differences in the percentage of readmissions in the 2 months (control group: 25.0 vs intervention group: 11.4, size of the percentage difference: − 54.4%, *p* = 0.041) and 6 months (control group: 42.2 vs intervention group: 24.3, size of the percentage difference: − 42.4%, *p* = 0.028) of follow up (Table 2).

As shown in Table 3 a high-intensity level of intervention was found in the study carried out by Rich and colleagues [30], with a total score of 28 out of 31 points. In this study, the home visits were scheduled earlier at the beginning of the post-discharge follow up compared to the other studies. They were planned to be performed within 48 h after hospital discharge but were performed most of the time within 24 h. This trial combined home visits and telephonic contacts between the members of the study team and the patients. The patients were seen at regular intervals and the communication was always open to provide advice to patients who required it. However, Rich and colleagues [30] did not report how often the team members performed the telephonic contacts. Thus, the authors were contacted via email to obtain this information, but they could not specify how long and often this telephonic contact had been.

**Table 3:** Qualitative assessment of intervention intensity

Study ID/ Length of intervention	Number of components in the pre-discharge phase^**a**^	Number of components in the post-discharge phase^**a**^	First visit at home or telephonic contact after hospital discharge^**b**^	Combination of home visit and other type of follow-up^**c**^	Number of scheduled home visits and/or telephone follow-up^**d**^	Duration of the intervention^**e**^	Availability of the health professional^**f**^	Total score of intervention intensity	Intervention Intensity level
**Clemson 2016** **[**28**]** At one month of intervention	6	3	1	3	2	1	1	16	Moderate intensity
**López Cabezas 2006** **[**29**]** At two months of intervention	6	4	1	1	2	2	2	18	Moderate intensity
At six months of intervention	6	4	1	1	4	2	2	20	Moderate intensity
At 12 months of intervention	6	4	1	1	2	3	2	19	Moderate intensity
**Rich 1995** **[**30**]** At two months of intervention	9	6	2	3	4	2	2	28	High intensity

**Notes:** ^a^One point was assigned for each TCM component used (Screening = 1, Staffing = 1, Maintaining Relationships = 1, Engaging Patients & Caregiver = 1, Assessing/Managing Risks & Symptoms = 1, Educating/Promoting Self-Management = 1, Collaborating = 1, Promoting Continuity = 1, Fostering Coordination = 1)

^b^First visit at home or telephonic contact after hospital discharge (First home visit or telephonic contact after 3 days of hospital discharge = 1, First home visits or telephone contact between 24 h to 3 days of hospital discharge = 2, First home visit or telephone contact within 24 h of hospital discharge = 3)

^c^Combination of home visit and other type of follow-up (Telephone follow-up or other type of follow-up without home visits = 1, Only home visits = 2, Home visits combined with telephone follow-up or other type of follow-up = 3)

^**d**^Number of scheduled home visits and/or telephone follow-up (1 = 1, 2–3 = 2, 4–5 = 3 and 6 or more = 4)

^**e**^Duration of intervention (30 days = 1, 31–180 days = 2, and 181–365 days = 3)

^**f**^Availability of the health professional (< 7 days or unable to determine = 1, 7 days = 2)

Finally, in order to compare the intensity scores of the interventions, three categories were created (Low intensity = 1–10 points, Moderate intensity = 11–21 points, High intensity = 22–31 points)

For the study of López Cabezas and colleagues [29], the intensity assessment was performed taking into account the three time periods of follow-up that they used, in order to see if there was a variation in the level of intensity between these periods. In general, a moderate-intensity level of intervention was found for the three follow-up periods. The total scores were 18, 20 and 19 points in respect to the 2, 6 and 12 months of follow up. This moderate-intensity level is explained by the number of telephonic contacts between the pharmacists and patients (monthly within the first 6 months of follow up and every two months for the remaining months of follow-up), the availability of the pharmacists to solve patient’s doubts at any time and the moderate number of TCM components applied (Table 3).

Clemson and colleagues [28] showed a moderate-intensity level of intervention with a total score of 16 points. They scheduled the home visits within the first week and additional visits were paid when it was required without previous scheduling. The home visits were combined with a telephonic contact every two weeks during the follow up time (Table 3).

Moreover, a survival curve for the probability of not being readmitted was estimated in two of the evaluated studies. That was the case of López Cabezas and colleagues [29] and Rich and colleagues [30], where after adjusting the model for predictors of readmission, a lower probability of readmission in the participants of the intervention group was observed (data not shown). Rich and colleagues [30] found statistically significant differences at three months of follow up (*p* = 0.03) and López Cabezas and colleagues [29] evidenced statistical significant differences at 12 months of follow up (*p* = 0.0095). Finally, regarding the presence of adverse events, these were not reported by the authors of the three studies.

#### Type of TCM components included in the interventions

Only the trial conducted by Rich and colleagues [30] applied all of the nine TCM components described by Hirschman and colleagues [31]. They used all nine components in the pre-discharge phase and six of seven components in the post-discharge phase. Regarding to the trial conducted by López Cabezas and colleagues [29], in total six TCM components were used in the pre-discharge and four in the post-discharge phase. The study carried out by Clemson and colleagues [28] also included in total six TCM components in the intervention. All of these were applied in the pre-discharge phase and three in the post-discharge phase (Table 4).

**Table 4:** Used TCM components of included studies

Study ID			Clemson 2016 [28]		López Cabezas 2006 [29]		Rich 1995 [30]	
TCM Component		Definition	Pre hospital discharge	Post hospital discharge	Pre hospital discharge	Post hospital discharge	Pre hospital discharge	Post hospital discharge
1	**Screening**^a^	Targets the key evidence-based risk factors from those who would benefit from the TCM intervention. According to Hirschman [31] the risk factors for eligible patients are: ≥ 5 active chronic conditions, a recent fall, deficits in basic activities of daily living (ADL), a diagnosis of dementia or poor performance on cognitive impairment screening tools, history of mental or emotional health problems and hospitalization within the past 30 days or ≥ 2 hospitalizations within the past six months.	X	^a^	X	^a^	X	^a^
2	**Staffing**^a^	Consists of the delivery and coordination of care is executed by the same master’s prepared advanced practice registered nurse (APRN), who assumes primary responsibility for the care of patients.	X^**b**^	^a^	X^**b**^	^a^	X^**b**^	^a^
3	**Maintaining Relationships**	Key feature of TCM to maintain and promote respectful and trusting relationships with patients and their family caregivers. This includes not only home visits and telephone calls, but also availability of the APRN or the health professional in charge of the intervention seven days a week.	X	X	X	X	X	X
4	**Engaging Patients and Caregivers**	Consists of the development and application of a discharge education and care plan in collaboration with the medical team, the patient and the caregivers. This plan includes the patient goals and preferences, among others.	X				X	
5	**Assessing/Managing Risks and Symptoms**	Comprehensive and targeted assessment to determine changes in the patient health status as well as a complete management of symptoms to prevent their onset or their risks.	X	X	X	X	X	X
6	**Education/Promoting Self-Management**	Involves the implementation of educational and behavioral strategies to meet the patients and caregivers learning needs related to an adequate and immediate response to the worsening of symptoms.			X	X	X	X
7	**Collaborating**	Refers to the furthering of consensus on the patients’ plan of care between patients and members of the healthcare team.					X	X
8	**Promoting Continuity**	Highlights the follow up of the patients by the same medical care team, in order to avoid interruption of the patients’ plan of care.	X	X	X	X	X	X
9	**Fostering Coordination**	Encourages the active communication between healthcare team and community-based practitioners, where the APRN in collaboration with patients, caregivers and team care members may identify the need for additional services.					X	X
**Total:**			6/9	3/7	6/9	4/7	9/9	6/7

^**a**^Since it is the same sample and the same staff as in the pre-discharge phase, these components are not needed to be used again after the hospital discharge. ^**b**^The intervention was carried out by other health professionals, such as occupational therapists [28], Pharmacists [29], and multidisciplinary team - including nurses among others health professionals [30]

### Secondary outcomes

Other outcome measures reported by Clemson and colleagues [28], showed that the intervention did not reduce difficulty with ADLs (ß = -0.17, 95% confidence interval CI = − 0.99-0.66) and participation (ß = -0.23, 95% CI = − 2.05-1.59). Additionally, López Cabezas and colleagues [29] and Rich and colleagues [30] assessed quality of life, but only Rich and colleagues observed a statistically significant improvement of this construct in the intervention group compared to the control group (*p* = 0.001).

Regarding the number of deaths, López Cabezas and colleagues found significant differences between the percentage of deaths of the control (29.7%) vs intervention (12.9%) group for the period of 12 months of follow-up (*p* = 0.017). Rich and colleagues did not observe significant differences in the assessment of this outcome, but observed differences in the percentage of deaths of the control group (12.1%) vs the intervention group (9.2%).

Moreover, these two studies performed a financial analysis, assessing if the reduction in hospital readmissions decreased the overall cost of care. Thus, López Cabezas and colleagues stated that participants in the intervention group evidenced savings of €578 per patient, while Rich and colleagues [30] showed $460 less per patient. None of the studies evaluated further outcome measures, related to the listed ones in the methods.

### Assessment of internal and external validity

The three included studies presented a low risk of bias related to the domain of randomization as well as to the domain of allocation concealment. Regarding the domain of incomplete outcome data, Rich and colleagues [30] and López Cabezas and colleagues [29] showed a low risk of bias, whereas Clemson and colleagues [28] exhibited a high risk of bias due to the 15 and 16% loss to follow-up in the intervention and control groups, respectively. Moreover, after evaluating the studies across the domain of blinding of outcome assessment, a low risk of bias was observed in Rich and colleagues [30] and Clemson and colleagues [28] trials. In the case of López Cabezas and colleagues [29], a high risk of bias was found, since this was an open clinical trial (Table 5). Clemson and colleagues [28] presented some limitations, such as the absence of a no-intervention control group comparison, or a possible contamination within the control group due to the option, that some participants of the control group were referred to occupational therapy after discharge. The trial of Rich and colleagues [30] was judged with high internal validity, whereas the other two trials were judged with medium internal validity.

**Table 5:** Assessment of internal and external validity

Study ID	Internal validity^**a**^				External validity^**b**^						
	Randomization	Allocation concealment	Blinding of outcome Measurement	Incomplete outcome data	Precision of effect estimates	Study design and Setting	Intervention and Control - assessment of performance bias	Outcome measurements, results and evaluation- assessment of detection bias	Indirectness: study population – assessment of selection bias	Directness of body of evidence	Rating of external validity
Clemson 2016 [28]	Low risk	Low risk	Low risk	High risk	+	+	(+)	(+)	(+)	(+)	(+)
López Cabezas 2006 [29]	Low risk	Low risk	High risk	Low risk	(+)	+	(+)	(+)	(+)	(+)	(+)
Rich 1995 [30]	Low risk	Low risk	Low risk	Low risk	(+)	+	(+)	(+)	(+)	(+)	(+)

^a^ The summary assessment of internal validity is presented as a description of the risk of bias in individual domains for each study according to Cochrane Risk of Bias Tool. ^b^The summary assessment of external validity was adapted from the Checklist of Bornhoft and colleagues [44] and it was evaluated as: **+** Matches completely/is completely fulfilled, **(+)** Matches incompletely but sufficiently/is only partly but sufficiently fulfilled, **−** Does not match or matches insufficiently/is insufficiently fulfilled

All included trials sufficiently fulfilled most of the external validity aspects. Therefore, our rating showed a medium external validity for the included trials. All studies matched completely the aspect of “Study design and Setting” and fulfilled incompletely but sufficiently most of the remaining external validity aspects defined by Bornhoft and colleagues [44] (Table 5). Specifically the aspect of “Precision of effect estimates” was completely fulfilled by Clemson [28], while by López Cabezas and colleagues [29] and Rich and colleagues [30] this aspect was only partly but sufficiently fulfilled (Table 5) [44].

## Discussion

Although several systematic reviews regarding transitional care for older people have been already conducted [14, 20], to our knowledge, this is the first systematic review on the use of TCM components addressing the geriatric patients (> 65 years old and with multi-morbidity), and all-cause readmission rate reduction. The present systematic review had as an objective to identify and summarize the different TCM components implemented in the included studies to guarantee safe transitions from hospital to community dwelling in order to reduce hospital readmissions in geriatric patients. Another objective was to recognize the Transitional Care Model components’ role and impact on readmission rates reduction. Addressing this very specific population – which will be an important issue due to the demographic change in the upcoming future – the findings of this systematic review provide valuable information that can provide guidance to health care professionals or to the development of evidence-based transitional care interventions. The increasing number of geriatric patients implies the utmost need to adapt the structures and methodologies of the current public health care systems. The diversity of professionals taking the lead in geriatric care is supported by the included three intervene studies: one being lead by occupational therapy (Australia), one conducted by pharmacy (Spain) and the last one in cardiology setting (US). This also demonstrate the diversity of the three international health care systems.

Although we realize that due to our established inclusion criteria, we only came up with three included studies – demonstrating the need for future studies with high quality, and larger sample sizes – the extracted information is very solid and of high research standards. Within the excluded studies are the basic studies conducted on the TCM assessment by Naylor and colleagues, since they did not meet the inclusion criteria related to participants age (> 65 years old), nor the intervention period [17, 24, 25]. Similarly, other studies were excluded for not meeting the eligibility requirements, e.g., Cao et al., 2017 and Bekelman et al., 2018, which did not meet the age criteria [47, 48]. With regard to “healthy aging” as proposed by the World Health Organization in 2015, all efforts have to be made for geriatric patients with multi-morbidity and chronic conditions to help them stay independent even in the context of care transitions [49, 50].

### Primary outcome: reduction of the readmission rate

This systematic review found meaningful differences between the three included trials, which are important for designing future trials and for the identification of relevant aspects in the improvement of transitional care in geriatric patients.

Aspects such as the length of the intervention were different between the three trials. The trial of Rich and colleagues [30] evidenced a successful reduction of the readmission rate at three months of intervention and follow up. For its part, Lopez Cabezas and colleagues [29] found this success in the reduction of the readmission rate at two and six months of intervention and follow up, but not at 12 months of intervention and follow up. A non-significant readmission rate was observed by Clemson and colleagues [28] after one month intervention, at the three months of follow up (Table 2). These different findings suggest that the length of the intervention as one aspect seems to influence the readmission rate. Future research is needed, to evidence the optimal length of transitional care for geriatric patients. Furthermore, the needed staff is being an economical issue for the public health care systems.

The included trials were rated in this systematic review as moderate- to high-intensity interventions of transitional care (Table 3). Based on these results, it is possible to hypothesize that the intensity level may have a relevant effect in reducing the readmission rate. Verhaegh and colleagues evidenced as a result of their meta-analysis that high-intensity interventions were associated with reduced short-term, intermediate-term and long-term readmissions [38]. They found a significant association between the first home visit within the first three days after hospital discharge and the reduction of short-term readmission rates. The results are related with the inverse relationship between early follow-up and risk of readmission already established by Hernandez and colleagues [51]. An explanation for these associations could be the characteristics of geriatric patients, who need a complex care, considering their high vulnerability and fragility. Particularly, for those patients who live alone, intensive supportive interventions after hospital discharge may play an important role in the prevention of hospital readmissions.

In relation to the multicomponent intervention approach, recent literature reviews evidenced a significant association between the number of transition components included in an intervention and the probability of success in the reduction of readmissions [20, 52–54]. Additionally, the research group of Burke inquired about the specific role of each component in reducing readmission rates. The component Cp5: Assessing / Managing Risk and Symptoms was the component most likely to reduce readmissions. An application of the components Cp9: Fostering Coordination and Cp6: Education/ Promoting Self-Management exhibited also a significant effect in reducing readmission rates [53]. Furthermore, these findings are in line with the existing research of Koelling and colleagues, who evaluated an educational component individually, evidencing less risk of rehospitalization in patients receiving the education intervention compared to patients receiving usual care [55].

### Cp 1: screening

All of the three trials performed this component due to the study design. However, geriatric patients should also be identified in the daily routine of the hospital. According to Greysen and colleagues [56] participants who are more fragile (poorer physical function, older age, suffering from multi-morbidity, impairment in activities of daily living, etc.) are the ones who tend to present higher readmission rates. There are evaluated geriatric assessments, e.g. ISAR score to identify these patients [57]. Beyond that, their special needs should be considered individually. De Wit and Schuurmans [58] suggested that this approach could lead to a slower deterioration in the condition of the patients and limited unplanned (re-)admissions.

### Cp 2: staffing

Although the traditional TCM uses advanced practice registered nurses to provide the hospital to community dwelling intervention, one positive aspect of our finding is that different professions seem to successfully implement the TCM model, which broadens the possible implementation process of this model. All three studies used different professions to implement their transitional care intervention. Trained occupational therapists, who aimed to provide patients with self-care skills that allow them to cope with daily living issues and return to their daily life activities, conducted the intervention in the trial of Clemson and colleagues [28]. Pharmacists carried out the intervention in the trial of López Cabezas and colleagues [29], who were experts on medicines. Thus, they were supportive in improving medication adherence as they provided education regarding medical doses, frequencies and number of dose intervals for the medical treatment of patients [59]. Rich and colleagues [30] used a multidisciplinary team and reported a significantly reduced readmission rate. These findings are in line to a recent study where particularly multidisciplinary network in heart failure management has been related to a reduction of rehospitalization, prolonged survival, and improved quality of life [60]. De Wit and Schuurmans [58] also call for a multidisciplinary collaboration across the different sectors.

It has to be taken into account that internationally there are different standards in the education of the healthcare team. For example, nurses or therapists are educated academically or non-academically, depending on the country of their education.

Finally, it could be possible that the impact of the contact given by the different professionals of the studies evaluated, could vary mainly because of the nature of the professions included (Occupational Therapists, Pharmacists and a multidisciplinary team), as well as by the uni- or multidisciplinary aspect of the work team. This difference may have had an impact not only on the desired outcomes but operationally on the transition components that could be applied according to the professions carrying out the interventions and follow-up. Therefore, it could be suggested that the joint work of a multidisciplinary team would have a greater impact on patient contact.

### Cp 3: maintaining relationships

This component was included in all three trials by caring for the patients and their family caregivers, both in the hospital and in the community dwelling. The healthcare professionals keep a relationship with patients and their caregivers through visits and telephone calls to prepare and accompany the patients during the implementation of the care plan and to meet their current and future needs. This approach is fairly consistent with the description of the TCM [31], in which maintaining relationships is a key feature. In addition, Le Berre and colleagues confirmed that this component leads to better adherence and disease control in geriatric patient, when the same person accompanies the patient in the transition from hospital to community dwelling [14]. Our findings support the importance of this component.

### Cp 4: engaging patients and caregivers

This component was applied only in two of the three trials [28, 30]. These trials engaged the people of their intervention groups in different ways.

Rich and colleagues [30] used a limited approach of patient engagement. Medication reconciliation by a geriatric cardiologist and modifications to the medications were made when necessary. These activities were carried out in cooperation with the patient, who additionally was required to keep a weight chart. In contrast, the patients in the trial of Clemson and colleagues [28] were asked to set client-centered goals. Additionally, only the research group around Clemson [28] mentioned the aspect of caregivers’ engagement, which was carried out depending on the availability of the patient’s family member but the authors did not report on the impact of their intervention on caregiver engagement.

It should be noted that a limited approach to caregiver engagement may reduce the impact of this component in the transition process, considering that care after hospital discharge generates a difficult burden on families [24]. If caregivers support and engagement can be included significantly in this component, however, this could relieve care giver burden substantially.

This component was included by the two trials, only in the setting of the hospital. However, in the course of the transition from one setting to the other, adjustments to the care plan may become necessary. Therefore, it is likely to be important to adjust the care plan also in the home environment. Thus, at home the patients and their family caregivers should be engaged again. In addition, it is worth mentioning to include the individual values and preferences in the care plan [31].

De Wit and Schuurmans [58] strongly encourage engaging geriatric patients to look after their own health. Likewise, a recent systematic review calls for integration of patients as full members of the care team; i.e. the patients should not only be informed, but also be empowered to participate [61]. However, Shearer and colleagues [62] stated that the well-defined empowerment intervention strategies were limited as well as not linked to theoretical frameworks. Therefore, it is recommended that future studies - designed to improve patient empowerment - should be better linked with established theoretical frameworks. In addition, these interventions should also take into account factors influencing hospital readmission, such as the discharge from hospital to patients own home when the patients depend on the help of someone else at home [63, 64]. It could be interesting to inquire about the correlation of such factors to the patients’ empowerment aspect, for instance, if engagement and empowerment of a patient for his own care could be less, when he has a greater expectation of family care, in order to identify the best way to involve patients and caregivers in the care plan. Another aspect could also be the impact on the health of the informal care, issues related to the older caregivers, such as physical and cognitive health problems. In this regard, a recent review have shown that the health of the older informal caregivers is at risk [65].

### Cp 5: assessing / managing risk and symptoms

This component was applied in all three trials. However, the implementation of this component was carried out differently in each study, based on the type of assessments and its goal. Clemson and colleagues [28] conducted measurements focused on a person’s functional ability to perform ADLs as well as on a person’s participation in life tasks and roles. They did not assess the symptoms of the disease nor did they evaluate other non-functional risks that may lead to the development of adverse events. Nonetheless, the assessment of the instrumental ADLs made it possible to draw conclusions indirectly about risk factors. López Cabezas and colleagues [29] and Rich and colleagues [30] applied this component more comprehensively, with the assessment of symptoms of the disease as well as risk factors for adverse events such as the side effects of the medication and the quality of life (QoL). It is noteworthy here that patients with a specific diagnosis (heart failure) were included in those two studies.

Especially in the geriatric population with the burden of multi-morbidity, it may be necessary to assess and manage the risk and symptoms individually. By looking at the domains of activity and participation, further undiscovered needs or dangers could be revealed. In general, Burke and colleagues [53] showed that this has been one of the components most associated with the reduction of readmission after discharge.

### Cp 6: education/promoting self-management

It became evident, that only the two trials [29, 30] that showed a reduction of readmission rate, applied an educational component. Both trials applied this educational component at pre- as well as at post-discharge. Jones and colleagues also pointed out the importance of this component and its implementation in both settings [66]. The educational component in the included trials was characterized by information and guidance related to illness, diet and medication. Lopéz Cabezas and co-workers [29] oriented their program to the social and cultural level of each patient. Furthermore, Jones and colleagues suggested an individualized educational approach [66].

Rich and colleagues affirmed that their educational component focused on intensive teaching and contributed to achieve significant readmission reductions. Particularly, since the educational component allowed to reinforce patient’s knowledge in the follow up, to guarantee adherence with medications and to provide information to recognize and manage persistent symptoms. These findings are in line with the research of Koelling and colleagues [55] and Burke and colleagues [53] who observed a lower risk of hospital readmission in patients receiving an education intervention. Furthermore, these results are in agreement with Hirschman and collaborators who mentioned that the educational component is important to reduce readmission rates, since education and self-management promotion allows to monitor, identify, understand, and answer to symptoms avoiding their exacerbation and worsening of the chronic condition [31].

### Cp 7: collaborating

This component was only included by Rich and colleagues [30]. It was applied at the hospital as well as in the community setting. The transition from hospital to community was developed collaboratively with a social worker and a member of the care team, facilitating a consensus on a plan of care. Thus, the collaborative work between multiple healthcare professionals who are not linked in the same network can provide a more complete approach of care [24]. As the WHO has called for person-centered and integrated care, integrating initiatives on service and organizational level seems mandatory to install the TCM component “collaboration”. This Integrated Care for Older People (ICOPE) approach of the WHO supports the collaboration components by integrating health and social care to improve the management of the geriatric persons [67].

### Cp 8: promoting continuity

Promoting continuity could help to prevent breakdowns in care across settings [31]. Several approaches to promoting continuity were used in the three trials [28–30]. The same people as in the hospital were also responsible for the patients in the community dwelling. A person of the care team could be reached by phone in case of problems or doubts at designated times. The first contact (telephone or home visit), which was made by the responsible healthcare professional was in a time interval known to the patient [61].

### Cp 9: fostering coordination

With respect to this component, Rich and colleagues [30] provided assistance in the case of emotional, social, but also economic or transportation problems during the discharge process. This points out, that not only an explicit standard for multidisciplinary communication is important, but also explicit standards for processes and systems are needed to ensure provider accountability, which would contribute to a successful transition [68]. Moreover, this component has been previously identified as being used frequently in interventions with an effect on the reduction of short term, intermediate term and long term readmissions [38]. In the present systematic review, the only trial that performed a long term intervention did not include this component and did not obtain a successful reduction of long term readmission [29]. Therefore, it is suggested that future studies should explore the effect of this component to the success of reducing long term readmissions.

In conclusion of the applied TCM components in our systematic review experiencing the “real” environment of geriatric patients will reveal barriers as well as supportive factors that can often not be detected from an inside-hospital view. This demonstrates the need of the community dwelling components of the TCM. Although not all components have been used by the three included trials, they addressed both care settings (hospital AND community) demonstrating the need of such an approach to reduce successfully the re-admission rate in a geriatric population.

Another interesting difference between the three trials is the nature of the TCM team. Looking at the history of the TCM, the focus is on a nurse-led intervention. However, in the included trials the configuration of the care team was not limited to this profession.

Regarding the multidisciplinary care team, the need for a team approach to improve the care of patients with chronic conditions has previously been emphasized [60]. In this case, only the trial carried out by Rich and colleagues [30] applied a multidisciplinary approach, the results of which were positive in reducing the readmission rates at the end of follow-up. These authors implemented more multidisciplinary care activities related to the management of heart failure, such as monitoring of symptoms and assessment of cardiovascular risk. The other two included trials [28, 29] performed the intervention using one health professional, and one of them [29] obtained successful results in the reduction of the readmission rate at two and six months of follow-up.

In conclusion, our systematic review demonstrated that the sum of the integrated components of the TCM is also responsible for a successful transition from the hospital to the community especially in geriatric patients. According to the results of this review, these factors are the intensity level and length of intervention, a multicomponent intervention approach and the specific role of each component, and the multidisciplinary nature of the care team. Future studies should focus on the optimal combination of these factors. In general, all of the nine defined TCM components by Hirschman and colleagues [31] were included across the three trials. Clemson and colleagues [28] and López Cabezas and colleagues [29] both included six of the nine components in their interventions, whereas Rich and colleagues [30] implemented all TCM components. In particular, in these studies more components were applied in the hospital settings than in the community dwelling, suggesting a potential imbalance in the inpatient setting support versus the in-home-follow-up support. However, it was not possible to establish from the three trials, whether this difference between how many components were used in hospital and at home may have had an effect on the successful transition to the community. Two trials affirmed that it was difficult to recognize which components were the most effective, since they administered a multifactorial intervention [29, 30]. According to the previous information, there is no clear evidence regarding which components were the most effective decreasing readmission rates. However, in light of the different results, the present systematic review attempts to evidence which components may have played a key role decreasing readmission rates.

### Secondary outcomes

There were also different results in relation to the secondary outcomes of interest.

In case of the QoL, only one of the evaluated trials [30] found a significant increase in the used QoL score. Rich and colleagues [30, 46] used a more specific instrument for their study population, the Chronic Heart Failure Questionnaire, which has been widely validated in older people with heart failure, and has shown adequate sensitivity in detecting clinically important changes over time as well as adequate scores for interpretability [30]. On the other hand, although López Cabezas and colleagues used a validated Spanish version of the EuroQol questionnaire [29], this questionnaire is not a specific instrument to assess QoL in people with heart failure, nor is it a questionnaire with items easy to interpret by the older population, especially with low cultural level, as discussed by López Cabezas and colleagues in their study. Apparently, these findings may be influenced by the instruments applied to measure this construct as well as to the characteristics of the study population. Regarding cost savings, both trials, Rich and colleagues [30] and López Cabezas and colleagues [29], showed lower costs for intervention vs usual care, where specifically Rich and colleagues suggest a long-term cost saving due to the multicomponent approach used. This is in line with other authors, who have also identified an overall reduction in the healthcare system costs due to transitional care interventions [69].

Clemson and colleagues [28] did not find improvements in their primary outcomes such as ADLs and participation in life roles and activities. Two other studies in this field, not included in this review, however found statistically significant improvements in the used measurement regarding ADLs [70, 71]. Apart from the fact that Clemson and colleagues [28] could not find any significant results in their trial, the importance of this domain seems to be proven. Future studies, which will be engaged with the optimal implementation of transitional care, should consider the domain of ADLs and participation.

Finally, it is important to highlight the need to conduct trials focused in the geriatric population over 65 years, which allow a better identification of the TCM role according to the care needs of this population. The results previously reported in the literature on the effects of the TCM, vary greatly, especially due to the variety of populations evaluated. For instance, the systematic review carried out by Coffey and colleagues evaluated studies that applied transition care, each with a specific study population such as new mothers, infants and children, adolescents, older people, among others. They observed mixed findings, in which the results of some studies varied in relation to the cost effectiveness, outcomes as the number of hospitalizations as well as the quality of life [72]. Added to this, in the present systematic review we observed a low number of trials conducted in the geriatric population over 65 years, evidencing a limited evaluation of the TCM in this population.

### Limitations and strength of this systematic review

In the present review, only three trials were included that met the precise inclusion criteria on which the present review was based. The still existing fragmentation in most public health care systems especially in geriatric patients’ needs growing realization to overcome this barrier. Our strict inclusion criteria with the special focus on geriatric patients could have excluded other studies with valuable information.

In addition, it was not possible to obtain a clear description of the control group conditions of each trial. As different care and discharge routine could have effects on the acceptance and implementation on the results of TCM implementation it was interesting to see that in all three studies the components of maintaining relationship and continuity was applied. One could hypothesize that in daily discharge and transitional routine being applied for the control group, especially these components of the TCM are not applied. It should be considered that these were carried out in countries with different health systems, where the standard hospital discharge procedure may vary.

Moreover, a gap related to the evaluation of the fidelity of interventions was evidenced, which did not allow this aspect to be addressed in the present review and could pose a bias on the results. Nevertheless, as we followed strictly the protocol with obtaining risk of bias we think that no fidelity information is needed with regard to our main objective. Even so, we consider the inclusion of fidelity assessment criteria in further trials to be relevant, especially in studies that evaluate transitional care interventions in geriatric populations (over 65 years old). Given that characteristics such as multi-morbidity, the application of interventions in multiple sites (Hospital and Home) and the complexity of these interventions (several components of the intervention, multidisciplinary team, among others) could limit the maintenance of the trials fidelity.

On the other hand, the strength of our review is the strict focus on the geriatric population, providing concrete information on the effects of multi-component interventions in reducing readmission in the geriatric population – individuals over 65 years of age with multi-morbidity. In view of the significant increase in this population in the upcoming years, effective and realistic approaches are needed to reduce the readmission rate of these highly vulnerable people. We therefore think that this systematic review will add valuable information not disease oriented but addressing a growing percentage of population putting the health care systems on the edge in the future. Furthermore, the health of the informal carer in this population needs to be taken into account as well. An additional strength of our study is – although we only included three trials – all trials had more than 50 participants in each trial arm, and were of good quality, strengthening our findings and providing a solid base for future research, and designing new transitional care intervention in the geriatric population.

## Conclusion

The findings of the present systematic review suggest that high intensity multicomponent, multidisciplinary interventions are likely to be effective reducing readmission rates and improving quality of life in geriatric patients, without increasing cost [29, 30]. Our systematic review underlines that components such as staffing, assessing and managing symptoms, educating and promoting self-management, maintaining relationships and fostering coordination seem to have an important role in reducing the readmission rate. This is of importance as educating and promoting self-management, maintaining relationship, and fostering coordination are not included in daily routine in the translation care process. These findings should be taken into account to strengthen healthcare in geriatric patients. In addition to the multicomponent nature of the intervention, its intensity represented as duration and frequency, as well as a multidisciplinary team of healthcare professionals increases the possibilities of obtaining positive outcomes. It is recommended to perform further investigations with an appropriate design, in order to better characterize the effects of the TCM components in the geriatric population. Finally, the finding that for the analysis of this systematic review, only three studies could be found, that included patients exclusively above 65 years of age, points to a need of further investigations addressing geriatric patients well above this age.

## Supplementary information

**Additional file 1.** MEDLINE Search strategy.

**Additional file 2.** Hand search in other sources.

**Additional file 3.** Parameters for the intervention intensity evaluation.

## Data Availability

The datasets used and analyzed during the current systematic review are available from the corresponding author on reasonable request.
