# Supplementary material for: Components of the transitional care model (TCM) to reduce readmission in geriatric patients: a systematic review
Source: BMC Geriatr. 2020 Sep 11;20:345. doi: 10.1186/s12877-020-01747-w (PMC7488657; doi:10.1186/s12877-020-01747-w)
Supplement: Supplementary file 1 — Additional file 1. MEDLINE Search strategy. [file 12877_2020_1747_MOESM1_ESM.pdf]

Additional file 1 - MEDLINE Search strategy

(((((aged[MeSH Terms]) AND ((((((((transitional care) OR continuity of patient care[MeSH Terms]) OR transition* AND of care) OR transitional care[MeSH Terms])) OR ((((((((caregiver well-being OR caregiver engagement))) OR ((patient engagement) OR (patient well-being))) OR patient education) OR caregiver education) OR family caregiver) OR continu* AND care)) OR ((((medication management) OR medication*) OR medication adherence) OR compliance))) AND (((((((((((patient readmission[MeSH Terms]) OR patient readmission) OR readmission*) OR readmission) OR rehospitalisation) OR rehospitalization) OR ((rehospitalisation) OR rehospitalization)) OR (((reduc* AND hospitalisation) AND reduc* AND hospitalization))) OR re-admission*) OR re-admission) OR re-admit*)))) AND (((((clinical trials as topic[MeSH Terms]) OR controlled clinical trials as topic[MeSH Terms])) OR controlled clinical trial[Publication Type]) OR randomized controlled trial[Publication Type]))) AND ("1994/01/01"[Date - Publication] : "3000"[Date - Publication])
