# Supplementary material for: Components of the transitional care model (TCM) to reduce readmission in geriatric patients: a systematic review
Source: BMC Geriatr. 2020 Sep 11;20:345. doi: 10.1186/s12877-020-01747-w (PMC7488657; doi:10.1186/s12877-020-01747-w)
Supplement: Supplementary file 2 — Additional file 2. Hand search in other sources. [file 12877_2020_1747_MOESM2_ESM.pdf]

Additional file 2 - Hand search in other sources

Type of resource	Source
**Other databases**	Catalog of the German National Library "Katalog der deutschen Nationalbibliothek" (https://portal.dnb.de/)<br>The International Clinical Trials Registry Platform (ICTRP) of the World Health Organization (http://www.who.int/ictrp/search/en/)<br>Registry of Clinical Trials of the United States National Library of Medicine (NLM) (https://clinicaltrials.gov/)<br>German Clinical Trials Register "Deutschen Register Klinischer Studien" (DRKS) (https://www.drks.de/drks_web/navigate.do?navigationId=start&messageDE=Home&messageEN=Home)<br>Chinese Clinical Trial Registry (ChiCTR) (http://www.chictr.org/)<br>Netherlands Trial Register (http://www.trialregister.nl/trialreg/index.asp)<br>EU Clinical Trials Register (https://www.clinicaltrialsregister.eu/ctr-search/search)<br>NIPH Clinical Trials Search (http://rctportal.niph.go.jp/en/)<br>Clinical Research Information Service (Cris) (https://cris.nih.go.kr/cris/en/index.jsp?mobile=)<br>The Brazilian Clinical Trials Registry "Registro Brasileiro de Ensaios Clínicos" (ReBEC) (http://www.ensaiosclinicos.gov.br/)<br>Cuban Registry of Clinical Trials "Registro Público Cubano de Ensayos Clínicos" (http://registroclinico.sld.cu/en)
**Internet searching**	National Association of Clinical Nurse specialists (https://nacns.org/)<br>My Aged Care (myagedcare.gov.au)<br>Transitional Care Management (transitionalcare-mgmt.com)<br>Centers for Medicare and Medicaid Services (cms.gov)<br>American Nurse Today (https://www.americannursetoday.com/page/2/?s=transitional+care)<br>The Joint Commission (https://www.jointcommission.org/toc_articles_and_publications.aspx)<br>Medicare Hospital Readmissions Reduction Program – RWJF (https://www.rwjf.org/en/library/collections/reducing-hospital-readmissions.html)<br>Chronic Care Management for Healthcare Providers (http://www.caresync.com/ccm/index.php)<br>The Colorado Health Foundation (http://www.coloradohealth.org/)<br>Council on Aging of Southwestern Ohio (http://www.help4seniors.org/)<br>Institute for Healthcare Improvement (IHI) (http://www.ihi.org/Pages/default.aspx)<br>ACC Quality Improvement for Institutions Program (https://cvquality.acc.org)<br>INTERACT- Training, Tools, Licensing and Resources (http://www.pathway-interact.com/interact-resources/publications/)<br>Next Step in Care (https://www.nextstepincare.org/search?q=transitional+care&submit.x=0&submit.y=0)

	Re-Engineered Discharge Project (Project RED) (https://www.bu.edu/fammed/projectred/publications.html)<br>The Care Transition Programs (https://caretransitions.org/our-publications/)<br>Health Science Center Jacksonville, University of Florida (https://hscj.ufl.edu/SearchResults.aspx?q=transitional%20care)
**Journals searching**	Geriatric Nursing (https://www.sciencedirect.com/journal/geriatric-nursing)<br>The American Journal of managed care (https://www.ajmc.com/)<br>Critical Care Nurse (http://ccn.aacnjournals.org/)<br>BMC Health Services Research (https://bmchealthservres.biomedcentral.com/)<br>Journal of Clinical Nursing (https://onlinelibrary.wiley.com/journal/13652702)<br>Journal of Professional Nursing (https://www.sciencedirect.com/journal/journal-of-professional-nursing)<br>Journal of Nursing and Care (https://www.omicsonline.org/nursing-care.php)<br>Journal of Gerontology & Geriatric Research (https://www.omicsonline.org/gerontology-geriatric-research.php)<br>BMC Nursing (https://bmcnurs.biomedcentral.com/)<br>Canadian Journal of Nursing Research (https://journals.sagepub.com/home/cjn)
