# Supplementary material for: Components of the transitional care model (TCM) to reduce readmission in geriatric patients: a systematic review
Source: BMC Geriatr. 2020 Sep 11;20:345. doi: 10.1186/s12877-020-01747-w (PMC7488657; doi:10.1186/s12877-020-01747-w)
Supplement: Supplementary file 3 — Additional file 3. Parameters for the intervention intensity evaluation. [file 12877_2020_1747_MOESM3_ESM.pdf]

Additional file 3 - Parameters for the intervention intensity evaluation

Parameter (1) Number of components in the pre-discharge phase, and Parameter (2) Number of component in the post-discharge phase: these two parameters were assessed assigning one point for each component implemented in the interventions at pre and post-discharge. Thus, the intervention was considered high-intensity when studies obtained at pre-discharge 9 points and 7 points at post-discharge, which meant that they implemented all the key TCM components proposed by Hirschman and colleagues (23).

Parameter (3) First visit at home or telephonic contact after hospital discharge: studies received the maximum score for this parameter, 3 points, when the first contact was performed within the first 24 hours, which meant that they followed the recommendations of the Promoting Continuity component (Cp 8) described by Hirschman and colleagues (23). 2 points were assigned, when this first contact was carried out within the first to third day, this value was adapted from Verhaegh and colleagues, see: Appendix A6 (34). Finally, when the first contact was established after three days of hospital discharge, a value of 1 point was assigned.

Parameter (4) Combination of home visits and other type of follow-up: according to Vedel and colleagues (39, 44) preferably, home visits should be combined with telephone follow-up or other types of follow-up in order to obtain positive effects. Thus, 3 points were assigned, when home visits were combined with telephone follow-up or other type of follow-up. Two points were given, when only home visits were carried out. One point was awarded, when just telephone follow-up or other type of follow-up was performed without home visits.

Parameter (5) Number of scheduled home visits and/ or telephone follow-up: studies obtained the maximum score, 4 points, when they performed six or more follow-ups; 3 points for four and five follow-ups; 2 points for two and three; and 1 point for one follow up. This criterion was established according to Verhaegh and colleagues, see: Appendix A6 (34).

Parameter (6) Duration of the intervention: This intensity parameter was also adapted from Verhaegh and colleagues, (34, 44) see: Appendix A6. Where 30 days= 1 point, 31-180 days= 2 points, and 181-365 days= 3 points.

Parameter (7) Availability of the health professional: two points were assigned, when the health professional availability was seven days a week, according with the description of the Promoting Continuity component (Cp 8) by Hirschman and colleagues (23). When the health professional's availability was less than seven days or was not clearly reported by the authors of the study, a value of 1 point was assigned.
